# Obesity: Its Causes and Treatment

**Published:** 1891-10-24

**Authors:** 


### OBESITY: ITS CAUSES AND TREATMENT.

The undue accumulation of fat in the subcutaneous tissues and around the internal organs is not only an inconvenience, but a diseased process. In perfect health, with proper diet and a reasonable amount of work, the percentage of fat ought not to exceed about five per cent. of the body weight (Burdach) and more than this indicates a departure from the healthy metabolism of the organism. It shows that the constructive forces are working in a wrong direction, and the energy which is used in the useless storing up of fat leaves the other tissues and organs in want. Consequently the adult man or woman who is "putting on flesh," is not generally to be congratulated. Fat people are less able to resist the attacks of disease or the shock of injuries and operations than the moderately thin. In ordinary every-day life they are at a decided disadvantage; their respiratory muscles cannot so easily act; their heart is often handicapped by the deposit on it; and the least exertion throws them into a perspiration. This last fact is curiously misunderstood; it is almost universally looked upon as an actual "melting" of the subcutaneous fat, and is considered to be nature's method of getting rid of the superfluity. But this is not correct, for in spite of its greasy appearance sweat only contains a trace of fatty matter, rarely more than ·01 per cent, and this comes, of course, from the cells of the sudoriparous glands and primarily from certain constituents in the blood. A person whose limbs and body are covered with adipose tissue is in the position of a man carrying a heavy burden and too warmly clothed. How great this burden can become may be seen by the effects of a sharp illness, which sometimes gets rid of this abnormal material at the rate of two or three stone in as many months, or even weeks. It is, in fact, the exertion causing increased circulation in the skin, and consequently increased filtration into the sweat glands which makes the perspiration, not the dissolving of the fat, which is practically untouched. In endeavouring to ascertain the reason why some people are corpulent and others not, we must realise at once that the condition is markedly hereditary. As one breed of pigs is noted for the ease with which it fattens, so with men. According to statistics collected by Bouchard this congenital predisposition can be traced in 33 per cent. of stout people, and Chalmers makes the proportion more than 50 per cent.

The amount of food and the proportions of its constituents have, doubtless, something to do with obesity. As to the actual quantity, it has often been observed that fat people eat less than thin ones; but when the process of fattening begins, it is almost always found that the subject is a great eater. (The mere statement of the individual must not be accepted in any case; people being notoriously unable to form a correct opinion on their own appetites.) When the abnormal activity of the fat-forming cells has fairly started, the condition persists obstinately, and the most moderate quantity of food leads to fresh deposit.

The metabolic changes are diminished in stout people; there is less waste and consequently less need for nourishment to replace it. It is therefore unjust to indiscriminately accuse these persons of over-eating.

Of the three great organic constituents of food it is most probable that all—proteids, carbohydrates and fats—may be converted into adipose tissue. The two non-nitrogenous can mutually replace each other in the economy, but there is no proof that carbohydrates alone can form fat. It has been thought by some physiologists that because cows manufacture such enormous quantities of fat-globules in their milk from food which is rich in carbohydrate material, that the latter is the source from whence the fat is derived. The fattening of pigs on potatoes, bran, meal, &c. (all containing a large amount of starch), has been adduced as an argument to prove this. Nevertheless it would seem that without proteid food the carbohydrates are useless for this purpose; and the total daily consumption of vegetable albumens by these animals is very great. Fats are to some extent undoubtedly deposited as fat; the carbon of the proteids is an important source of part of the complex molecule, whilst the carbohydrates supply the necessary hydrogen and oxygen. A diet of proteids with carbohydrates or fats is therefore more fattening than a more non-nitrogenous one. Slight excess of proteid food (as in those who eat meat twice daily), with large quantities of pastry, bread, potatoes and other starchy or saccharine food is the diet most conducive to fat formation.

*Diminished mental activity* is both a cause and a consequence of corpulency. People whose circumstances are too easy, and those whose intellects are clouded or apathetic (as in dementia, &c.), frequently become stout. The dulling effect of alcohol upon the nervous system is probably the chief reason why beer drinkers so often get fat. Other alcoholic drinks do not cause this so readily, partly because they cannot generally be indulged in to so great an amount, partly because the absorption and elimination is quicker than in the more dilute form, and partly, perhaps, because ale and stout, except in extreme cases of indulgence, are less likely to interfere with the digestive organs than spirits and wines. It should be remembered, however, that all achoholic drinks tend to diminish metabolism, and consequently the storage of food as adipose tissue.

Troubles, especially petty worries and annoyances, make for leanness. Alcohol in moderation, by counteracting anxious thought, may prevent this. The emaciation of the so-called "cancerous cachexia" is no doubt chiefly the result of worry and apprehension. Anything which quiets or amuses the mind tends to produce corpulency, as exemplified in the proverb "Laugh and grow fat."

*Exercise* must next be considered. The easy-going man is too frequently lazy and unwilling to use his muscles; the fidgetty man is continually "on the go"; the one gets stout, the other thin; but is it not the temperament rather than the movement which brings about this result? Nevertheless it is quite obvious that the more active a part is the more nutriment it requires, and the greater is the supply of blood; consequently work of the brain and muscles directs the metabolic force of the body to these tissues, and lessens the chance of undue activity of the fat-forming cells.

We may, therefore, sum up the causes of obesity thus: (1) Hereditary predisposition; (2) over-eating is a most important *exciting* cause, but when once started the amount is of less consequence; (3) abnormal admixture of food stuffs; (4) mental conditions; (5) want of exercise of the nervous and muscular systems; (6) alcohol (in certain forms especially).

We may condense this summary again by saying that undue accumulation of fat is owing to an abnormal distribution of the energy which guides the formative (anabolic) metabolism of the body. To treat the condition this force must be guided into the right channels.

(*To be continued.*)

